# An artificial EMG generation model based on signal-dependent noise and related application to motion classification

**DOI:** 10.1371/journal.pone.0180112

**Published:** 2017-06-22

**Authors:** Akira Furui, Hideaki Hayashi, Go Nakamura, Takaaki Chin, Toshio Tsuji

**Affiliations:** 1 Department of System Cybernetics, Graduate School of Engineering, Hiroshima University, Higashi-Hiroshima, Japan; 2 Department of System Cybernetics, Institute of Engineering, Hiroshima University, Higashi-Hiroshima, Japan; 3 Robot Rehabilitation Center in Hyogo Rehabilitation Center, Kobe, Japan; 4 Department of Orthopedic Surgeon, Hyogo Rehabilitation Center Hospital, Kobe, Japan; Universita degli Studi di Pisa, ITALY

## Abstract

This paper proposes an artificial electromyogram (EMG) signal generation model based on signal-dependent noise, which has been ignored in existing methods, by introducing the stochastic construction of the EMG signals. In the proposed model, an EMG signal variance value is first generated from a probability distribution with a shape determined by a commanded muscle force and signal-dependent noise. Artificial EMG signals are then generated from the associated Gaussian distribution with a zero mean and the generated variance. This facilitates representation of artificial EMG signals with signal-dependent noise superimposed according to the muscle activation levels. The frequency characteristics of the EMG signals are also simulated via a shaping filter with parameters determined by an autoregressive model. An estimation method to determine EMG variance distribution using rectified and smoothed EMG signals, thereby allowing model parameter estimation with a small number of samples, is also incorporated in the proposed model. Moreover, the prediction of variance distribution with strong muscle contraction from EMG signals with low muscle contraction and related artificial EMG generation are also described. The results of experiments conducted, in which the reproduction capability of the proposed model was evaluated through comparison with measured EMG signals in terms of amplitude, frequency content, and EMG distribution demonstrate that the proposed model can reproduce the features of measured EMG signals. Further, utilizing the generated EMG signals as training data for a neural network resulted in the classification of upper limb motion with a higher precision than by learning from only measured EMG signals. This indicates that the proposed model is also applicable to motion classification.

## Introduction

Surface electromyogram (EMG) signals obtained from the skin surface represent the action potential generated from muscle fibers constituting each motor unit, and reflect muscle activation levels. Many studies have therefore investigated their applicability in areas such as rehabilitation, prosthesis control, and motion analysis [1–5].

However, to apply the EMG signals in such areas, appropriate feature extraction for EMG is needed. The EMG signals can be assumed to be stochastic processes with amplitudes that vary with muscle activity [6, 7]. Hence, attempts to extract the EMG signal features have been conducted by modeling their stochastic characteristics [6–13]. For example, De Luca [8] derived EMG signal features such as the mean rectified value, the root mean square value, and the variance of the rectified signals from the stochastic properties of the inter-pulse interval and the motor unit action potential train (MUAPT). This was done based on a mathematical model derived with the underlying assumption that EMG signals are expressed as the sum of MUAPTs. Hogan and Mann [6, 7] modeled the relationship between muscle force and EMG signals based on a Gaussian white noise process with a zero mean and variance nonlinearly depending on muscle force. Inspired by this underlying concept, Hayashi *et al*. [13] expanded Hogan and Mann’s model by assuming that signal-dependent noise [14, 15] is superimposed according to muscle activation levels onto the EMG signal variance, resulting in an expression of the variance as a random variable following an inverse gamma distribution.

Mathematical EMG models such as these can also be applied to artificial EMG signal generation. A generation model that produces artificial EMG signals from an arbitrary characteristic given as input can be implemented as the inverse model of the feature extraction model. Artificial EMG generation methods based on physiological processes have been proposed and developed in previous studies [16–19]. For example, Farina *et al*. [16] proposed an artificial EMG generation model based on analytical derivations in EMG signals with cylindrical description of the volume conductor. Further, Person *et al*. [17] generated synthetic EMG signals using the summation of the firing patterns of action potentials.

One of the practical applications of artificial EMG signals is their use as training data for EMG classification based on machine learning, which enables a reduction of the burden of data collection and improvement of generalization capability. However, EMG signals are often measured during strong muscle contraction, in which signal-dependent noise superimposed according to the muscle activation levels cannot be ignored. In such situations, an artificial EMG generation model should also include the superimposed noise. However, previous artificial EMG generation methods, which were based on physiological processes, never considered this signal-dependent noise. In addition, these conventional methods tend to be cumbersome for generating EMG signals because they consist of many physiological parameters that have to be calibrated. As a result, they have not been applied to motion classification.

This paper proposes an artificial EMG signal generation model based on signal-dependent noise. In the proposed model, an EMG variance value is assumed as a random variable and is generated from a probability distribution with a shape determined by the commanded muscle force and signal-dependent noise. Artificial EMG signals are then generated by multiplying the variance value and a white Gaussian noise that passed through a shaping filter, thereby enabling the representation of artificial EMG signals with signal-dependent noise superimposed according to the muscle activation levels. In addition, the proposed model can generate artificial EMG signals simply by setting a few variance distribution parameters, which can be easily estimated from measured EMG signals.

## Materials and methods

### Model structure

**Fig 1:**
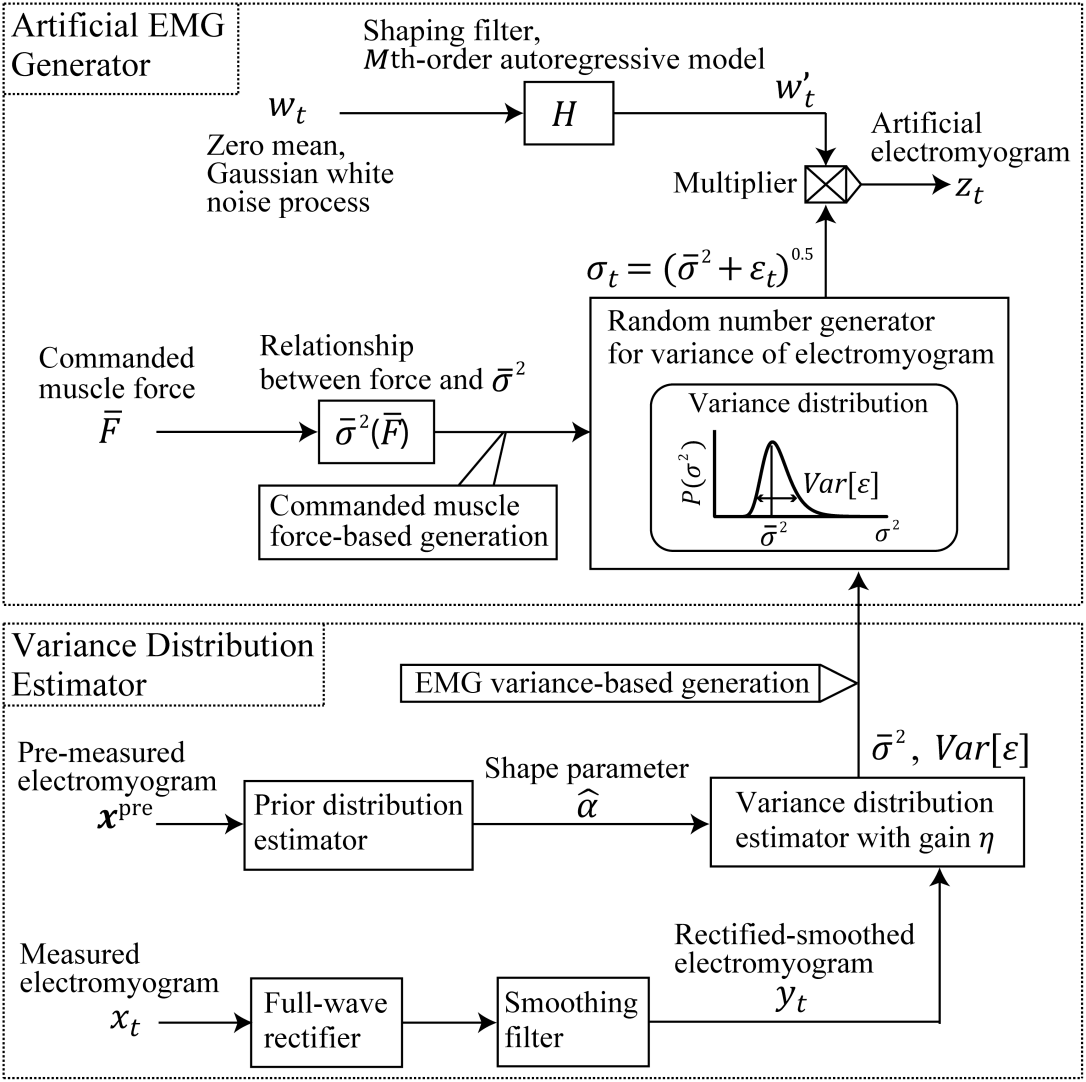
Overview of the proposed model. The model expresses an artificial EMG signal *z*_*t*_ at *t*, based on a process involving white Gaussian noise $w_{t}^{\prime }$ passed through a shaping filter *H* and variance $\sigma_{t}^{2}$. Variance $\sigma_{t}^{2}$ is the value at *t* of a random variable *σ*^2^ having a distribution determined by a commanded muscle force component of variance ${\bar{\sigma }}^{2}$ and signal-dependent noise *ε* according to the commanded muscle force $\bar{F}$.

Fig 1 gives an overview of the proposed model, which expresses an artificial EMG signal *z*_*t*_ at time *t* based on a process involving white Gaussian noise $w_{t}^{\prime }$ passed through a shaping filter *H* and variance $\sigma_{t}^{2}$. Variance $\sigma_{t}^{2}$ is the value at *t* of a random variable *σ*^2^ having a distribution determined by a commanded muscle force component of variance ${\bar{\sigma }}^{2}$, derived from Eq (1), and signal-dependent noise *ε* according to the commanded muscle force $\bar{F}$.

The relationship between $\bar{F}$ and $\bar{\sigma }$ can be expressed as

$$\begin{array}{c} \bar{\sigma }=k{\bar{F}}^{a}, \end{array}$$ (1)

where *k* and *a* are constants that can be experimentally estimated [6, 7]. Then, *σ*^2^ is represented by the sum of ${\bar{\sigma }}^{2}$ and *ε*:

$$\begin{array}{c} \sigma^{2}={\bar{\sigma }}^{2}+\varepsilon . \end{array}$$ (2)

Assuming that *ε* is a random noise with a zero mean, the mean *E*[*σ*^2^] and variance *Var*[*σ*^2^] of *σ*^2^ are calculated as follows:

$$\begin{array}{ccc} E[\sigma^{2}] & = & E\left[{\bar{\sigma }}^{2}\right]+E\left[\varepsilon \right]={\bar{\sigma }}^{2}, \end{array}$$ (3)

$$\begin{array}{ccc} Var[\sigma^{2}] & = & E\left[{\left(\sigma^{2}-{\bar{\sigma }}^{2}\right)}^{2}\right]=Var\left[\varepsilon \right]. \end{array}$$ (4)

Considering that *σ*^2^ > 0, the inverse gamma distribution IG(*α*, *β*) is chosen as the distribution of *σ*^2^ [13]:

$$\begin{array}{c} P\left(\sigma^{2}\right)=\text{IG}\left(\sigma^{2};\alpha ,\beta \right)=\frac{\beta^{\alpha }}{\Gamma (\alpha )}{\left(\sigma^{2}\right)}^{-\alpha -1}e^{-\frac{\beta }{\sigma^{2}}}, \end{array}$$ (5)

where *α* and *β* are parameters that determine the inverse gamma distribution and are referred to as the shape parameter and the scale parameter, respectively [20]. The relationships between [*α*, *β*] and the mean and variance of *σ*^2^ are expressed as follows:

$$\begin{array}{ccc} {\bar{\sigma }}^{2} & = & E\left[\sigma^{2}\right]=\frac{\beta }{\left(\alpha -1\right)}, \end{array}$$ (6)

$$\begin{array}{ccc} Var[\varepsilon ] & = & Var\left[\sigma^{2}\right]=\frac{\beta^{2}}{{\left(\alpha -1\right)}^{2}\left(\alpha -2\right)}. \end{array}$$ (7)

The artificial EMG signal *z*_*t*_ can be defined as the product of $w_{t}^{\prime }$ and random number series *σ*_*t*_, which is generated from the inverse gamma distribution determined by the mean ${\bar{\sigma }}^{2}$ and the variance *Var*[*ε*]:

$$\begin{array}{c} z_{t}=\sigma_{t}w_{t}^{\prime }, \end{array}$$ (8)

where $w_{t}^{\prime }$ is the Gaussian noise process, which has the same power spectrum as stationary EMG signals, and is generated from the following shaping filter based on an *M*th-order autoregressive (AR) model:

$$\begin{array}{c} w_{t}^{\prime }=\sum_{j=1}^{M}\;a_{j}w_{t-j}^{\prime }+\sqrt{v}w_{t}, \end{array}$$ (9)

where *w*_*t*_ is white Gaussian noise with mean = 0 and variance = 1, *v* is the estimated variance of error, and *a*_*j*_ (*j* = 1, ⋯, *M*) is the coefficient of the AR model. Because these AR parameters are estimated by normalized EMG signals with variance = 1, $w_{t}^{\prime }$ becomes Gaussian noise with mean = 0 and variance = 1.

The above procedure is the general form for generating artificial EMG containing signal-dependent noise superimposed according to the commanded muscle force (expressed as the commanded muscle force-based generation in Fig 1). However, this procedure requires the estimation of the parameters in Eq (1) that change depending on the skin condition and the location of the electrodes for each subject. Therefore, this paper proposes another approach called EMG variance-based generation. The proposed approach uses rectifying and smoothing EMG signals by directly estimating the distribution of *σ*^2^ using a small number of EMG samples recorded in advance.

### EMG variance-based generation

Hayashi *et al*. assumed that EMG signal *x* follows a Gaussian distribution with a mean of zero and a variance that follows an inverse gamma distribution. Consequently, they proposed an approximate estimation method for the mean and variance of variance that utilizes the property of rectified-smoothed EMG signals [13]. Unlike [13], we propose a new method to determine ${\bar{\sigma }}^{2}$ and *Var*[*ε*] by proportional modulation with an introduced gain *η*:

$$\begin{array}{c} {\bar{\sigma }}^{2}={\left(\frac{1-\sum_{i=0}^{N-1}\;a_{i}}{\sum_{i=0}^{N}\;b_{i}}\right)}^{2}\;\frac{\pi }{2}\eta^{2}E{\left[y_{t}\right]}^{2}, \end{array}$$ (10)

$$\begin{array}{c} Var\left[\varepsilon \right]=\frac{{\left({\bar{\sigma }}^{2}\right)}^{2}}{\left(\hat{\alpha }-2\right)}, \end{array}$$ (11)

where *a*_0_, *a*_1_, ⋯, *a*_*N*−1_ and *b*_0_, *b*_1_, ⋯, *b*_*N*_ are the coefficients of an *N*th-order low-pass filter, *η* is the gain for proportional variance modulation, *y*_*t*_ is a rectified-smoothed EMG signal at *t*, *E*[*y*_*t*_] is the expectation of *y*_*t*_, and $\hat{\alpha }$ is the shape parameter of variance distribution. From Eqs (3) and (4), the estimated values of ${\bar{\sigma }}^{2}$ and *Var*[*ε*] correspond to the mean *E*[*σ*^2^] and variance *Var*[*σ*^2^] of *σ*^2^, respectively. *Var*[*σ*^2^] can therefore be estimated from ${\bar{\sigma }}^{2}$ by setting $\hat{\alpha }$ in advance. $\hat{\alpha }$ is fixed and is estimated prior by maximizing the marginal likelihood of pre-measured EMG dataset ***x***^pre^ using the steepest descent method. Note that the proportional gain *η* modulates the EMG variance; thus, in the case of *η* = 1, the proposed model generates artificial EMG signals to reproduce the variance distribution of the measured signals.

As stated above, ${\bar{\sigma }}^{2}$ and *Var*[*ε*] can be estimated and modulated using the rectified-smoothed signal *y*_*t*_, shape parameter $\hat{\alpha }$, and proportional gain *η* based on Eqs (10) and (11).

### Experiments

#### Ethics statement

This study was approved by the Human Research Ethics Committee of the Hyogo Institute of Assistive Technology. All subjects were told the aim of the experiments and provided written informed consent before participating in the trial. The individual in this manuscript has given written informed consent (as outlined in PLOS consent form) to publish these case details.

#### Subjects

Ten healthy young adults (males, age range: 22–24 years; mean age: 21.8 ± 1.0 years) and four healthy young adults (males, age range: 21–23 years; mean age: 22.6 ± 0.8 years) were recruited in Experiment 1 and Experiment 2, respectively. Both experiments were conducted in Hyogo Rehabilitation Center from August 2015 to January 2016. All subjects were right-handed and were included on the basis of the following criteria: no previous physical, neurological, or sensory disorders, no medication that might influence their muscle activity, and no history of intense exercise in the previous 24 hours.

#### Experiment 1: Evaluation of generated artificial EMG signals

We conducted an evaluation experiment for artificial EMG signals generated using the proposed model. In the experiment, we first measured the EMG signals during constant isometric contraction of the biceps brachii of ten healthy subjects. By using the measured EMG signals, the variance distribution parameters ${\bar{\sigma }}^{2}$ and *Var*[*ε*] and the parameters for the shaping filter *H* were estimated. Artificial EMG signals were then generated based on the estimated parameters. Further, the accuracy of the generated signals was evaluated by comparing them with the measured EMG signals.

**Fig 2:**
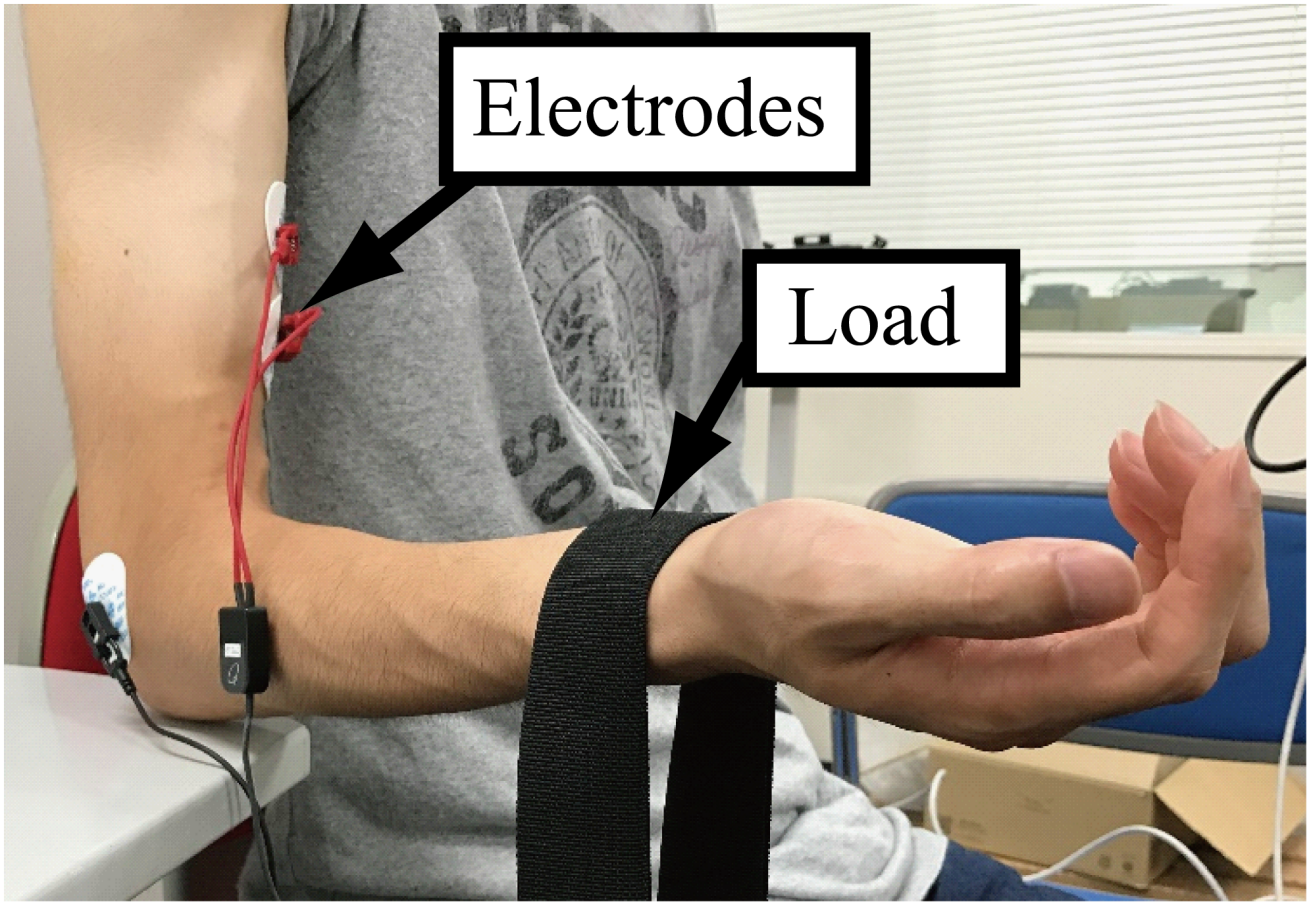
Scene of the EMG recording. The subjects were seated with the right upper arm pointing downward, the right forearm bent forward to the horizontal, and the palm turned upward. EMG signals were recorded from a pair of electrodes attached to the biceps brachii while the subjects were weighted with a load on the right wrist and maintained the right elbow on a desk.

For EMG signal recording, the subjects were seated, with the right upper arm pointing downward, the right forearm bent forward to the horizontal, and the palm turned upward (Fig 2). EMG signals were recorded using a pair of electrodes attached to the skin surface of the biceps brachii at a sampling frequency of 1000 Hz while the subjects were weighted with a load hanging vertically on the right wrist with the elbow on a desk (Fig 2). The subjects were instructed to maintain the posture for 10 seconds with the elbow at 90°. The load weight was varied through values of 500, 1000, 1500, and 2000 g, and one trial was conducted for each load weight. The range of these load weights was selected on the basis of the following criteria: it appears in everyday activities, subjects do not feel muscle fatigue, and the differences in the muscle activation levels can be acquired clearly. The latter five-second part of the ten seconds of recorded data was used for comparison and variance distribution estimation. A multi-telemeter system (NIHON KOHDEN, WEB-5000, high-frequency cutoff: 100 Hz, low-frequency cutoff: 5.4 Hz) was used for measurement.

To evaluate the reproducibility of the proposed model with respect to the measured EMG signals, the estimation of variance distribution was conducted for each load weight. The model order of the shaping filter was determined as *M* = 20 based on the Bayesian information criterion (BIC) [21], and the estimated variance of error *v* and the model coefficients *a*_*j*_ were determined using the Burg method [22]. The proportional gain was determined as *η* = 1.0, meaning that the variance modulation was not applied. A second-order Butterworth low-pass filter (cutoff frequency: 1 Hz) was used to smooth the EMG signals. Generation of artificial EMG signals using estimated parameters was also conducted, with ten trials for each load weight. Tanizaki’s method [23] and Box–Muller’s method [24] were used to generate inverse gamma and Gaussian random numbers, respectively.

The accuracy of the artificial EMG generated was evaluated in terms of average amplitude, frequency component, and kurtosis of EMG distribution. In general, the amplitude and the frequency component are the important features of an EMG signal. Kurtosis is the fourth central moment of distribution, and was utilized to evaluate the influence of the variation in variance on the shape of the EMG distribution. The average amplitude was determined from the average value of the rectified and smoothed signals. Further, the absolute percentage errors in the average amplitudes between the measured and artificial EMG signals were calculated for each load weight. With respect to the frequency component, the power spectrum densities of the measured and artificial EMG signals were calculated using the *M*′th-order AR model as follows:

$$\begin{array}{c} P\left(f\right)=\frac{v^{\prime }}{|1-\sum_{j=1}^{M^{\prime }}\;a_{j}^{\prime }e^{-i2\pi jf}{|}^{2}}, \end{array}$$ (12)

where *f* is frequency, $a_{j}^{\prime }\left(j=1,\cdots ,M^{\prime }\right)$ is the AR coefficient, and *v*′ is the estimated variance error. The model order *M*′ was determined as *M*′ = 20 based on the BIC. $a_{j}^{\prime }\left(j=1,\cdots ,M^{\prime }\right)$ and *v*′ were determined using the Burg method. The correlation coefficients in the power spectrum densities between the measured and artificial EMG signals were then calculated. Finally, the kurtosis of the EMG distribution was calculated for each load weight. Note that kurtosis is a measure of the tailedness of a probability distribution. The sample kurtosis for a univariate random process $x={\left\{x_{n}\right\}}_{n=1}^{N}$ can be calculated as follows:

$$\begin{array}{c} K=\frac{1}{N}\frac{\sum_{n=1}^{N}{\left(x_{n}-\bar{x}\right)}^{4}}{s^{4}}-3, \end{array}$$ (13)

where $\bar{x}$ and *s* are the mean value and standard deviation of ***x***, respectively. The root mean square error (RMSE) in the kurtosis between the measured and the artificial EMG signals was then calculated in all trials as follows:

$$\begin{array}{c} \text{RMSE}=\sqrt{\frac{1}{T}\sum_{t=1}^{T}{\left(K_{t}-\hat{K_{t}}\right)}^{2}}, \end{array}$$ (14)

where *T* is the number of trials (*T* = 10) for each load weight, and *K*_*t*_ and $\hat{K_{t}}$ are the sample kurtosis of the measured and the artificial EMG signals at trial *t*, respectively.

In the proposed model, the shape parameter $\hat{\alpha }$ and the parameters of the shaping filter need to be set in advance of artificial EMG generation using pre-measured EMG signals for each subject. Hayashi *et al*. [13] assumed that the shape parameter is a constant within an individual regardless of muscle activation levels. However, this assumption has not been verified experimentally. Therefore, because the muscle activation level on the pre-measured EMG signals can affect the generation accuracy of the proposed model, generation and evaluation of the artificial EMG signals were conducted by changing the source of the preset parameters. These preset parameters were set for each subject using EMG signals recorded in advance under each load weight.

For comparison, artificial EMG signals were also generated based on the Hogan and Mann’s model [6, 7], and evaluated. The major differences between the method based on the Hogan and Mann’s model and the proposed method is that EMG variance is handled as a constant and is estimated using maximum likelihood estimation in the former method. Finally, artificial EMG generation based on the proposed model with the variance modulation by the proportional gain was conducted to evaluate its generation accuracy. The EMG signals recorded under a 1000 g load were set as the reference, and the gain *η* in Eq (10) was defined as follows:

$$\begin{array}{c} \eta =\frac{f_{w}}{f_{1000}}, \end{array}$$ (15)

where *f*_*w*_ is the muscle force of the biceps brachii at load weight *w* = 500, 1000, 1500, 2000 g, and is calculated from each load weight following the procedure adopted by Hayashi *et al*. [13], with the body weight and the length from the elbow axis to the ulnar styloid. Note that by using this proportional gain, the artificial EMG signals at each load weight were generated only from the measured signals under a 1000 g load. These generated EMG signals were evaluated by comparing the measured EMG signals at each load weight. We compared the average value of the ten subjects in each index among the proposed method, the constant variance-based method, the and proposed method with the variance modulation. In this comparison, the shape parameter $\hat{\alpha }$ and the parameters of the shaping filter were set for each subject using the pre-measured EMG signals under a 2000 g load, and they were set in common throughout the comparison.

#### Experiment 2: Motion classification

**Fig 3:**
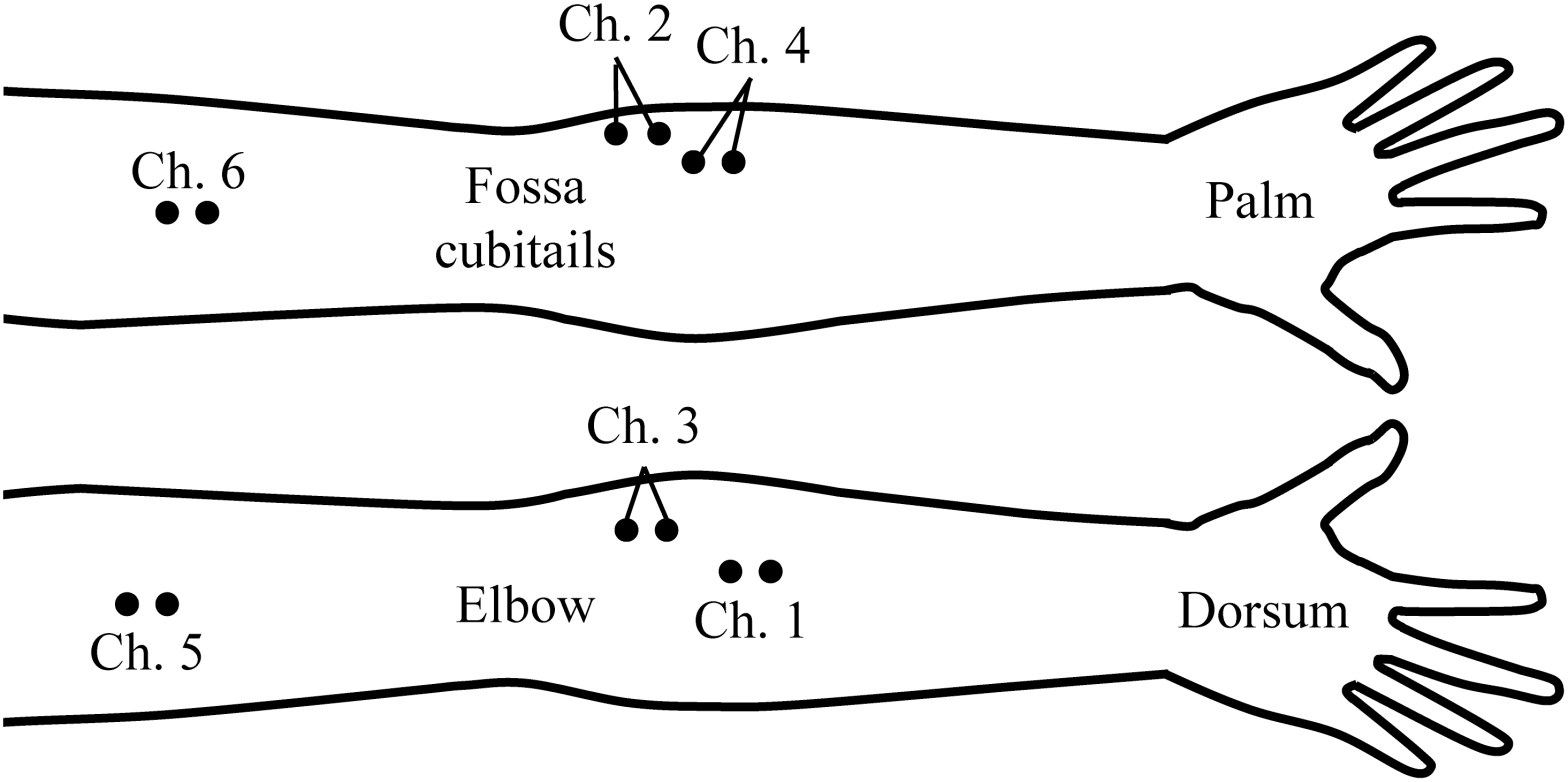
Location of the electrodes. EMG signals were recorded using six electrodes (*L* = 6: Ch. 1: extensor carpi ulnaris; Ch. 2: flexor digitorum profundus; Ch. 3: extensor digitorum; Ch. 4: flexor carpi ulnaris; Ch. 5: triceps brachii; Ch. 6: biceps brachii) at a sampling frequency of 1000 Hz.

To evaluate the applicability of the proposed model to motion classification, a classification experiment was conducted in which the generated EMG signals were utilized as training data for a neural network. This experiment was conducted on four healthy subjects (Subjects A–D). EMG signals were recorded using six electrodes (*L* = 6: Ch. 1: extensor carpi ulnaris; Ch. 2: flexor digitorum profundus; Ch. 3: extensor digitorum; Ch. 4: flexor carpi ulnaris; Ch. 5: triceps brachii; Ch. 6: biceps brachii) at a sampling frequency of 1000 Hz (Fig 3). The subjects performed six motions (*C* = 6): flexion, extension, supination, pronation, hand open, and hand grasp.

The EMG measurement system and the parameters for the smoothing process were the same as in the evaluation experiment. Prior to the experiment, pre-measurement of EMG signals during a maximum voluntary contraction (MVC) was conducted for each motion. The channel corresponding to the agonist muscle in each motion was then determined as follows:

$$\begin{array}{c} m=\underset{l\;\in \;\{1,\cdots ,L\}}{\text{arg}\;\text{max}}E_{t}\left[{\hat{y}}_{t}^{\left(l\right)}\right], \end{array}$$ (16)

where ${\hat{y}}_{t}^{\left(l\right)}$ is a pre-measured rectified-smoothed EMG signal of channel *l* at time *t* and $E_{t}\left[{\hat{y}}_{t}^{\left(l\right)}\right]$ is the expectation of ${\hat{y}}_{t}^{\left(l\right)}$ regarding *t*. The parameter $\hat{\alpha }$ and parameters of the shaping filter were also calculated from the pre-measured EMG signals for each subject.

**Fig 4:**
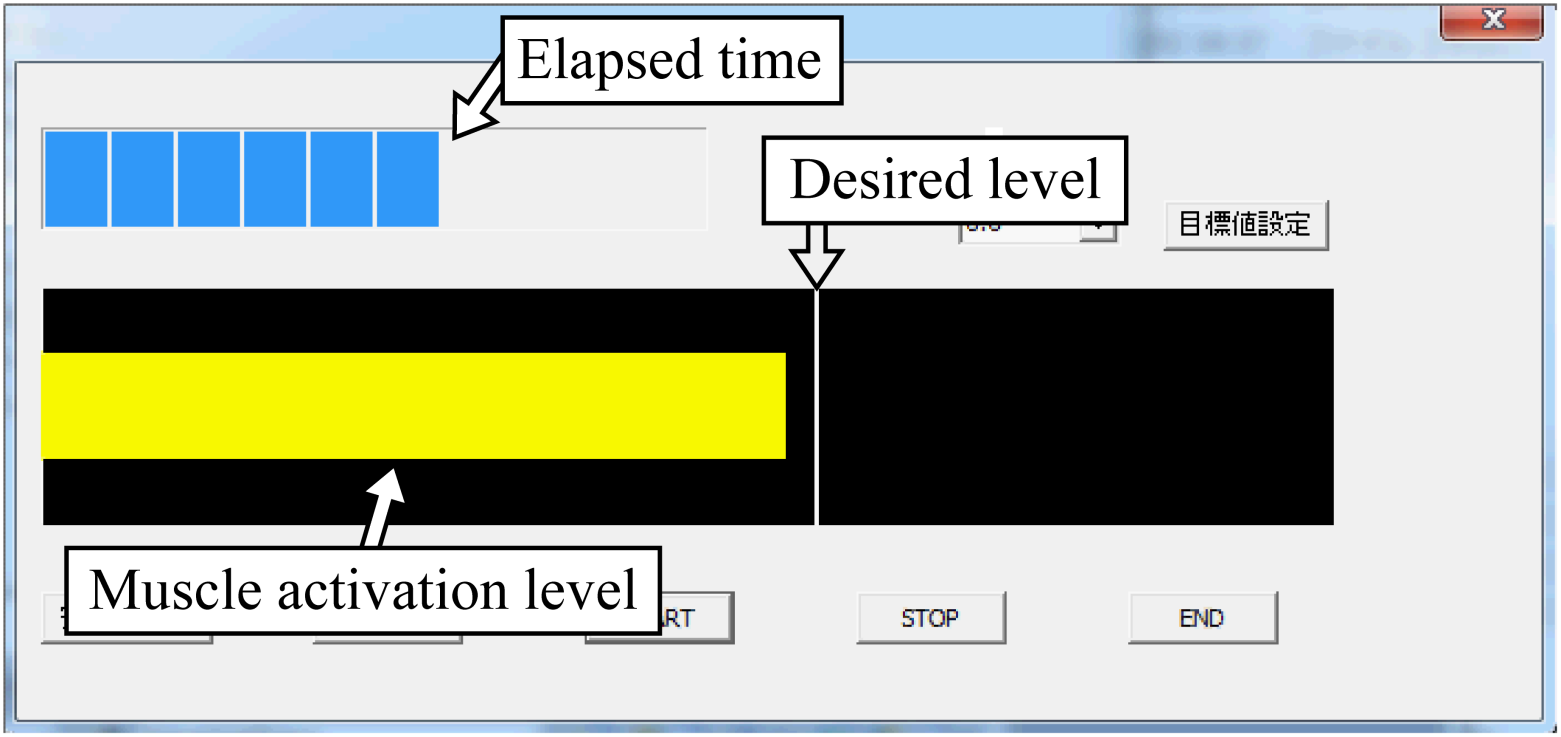
Screenshot of the EMG measurement system. The bar graph shows the muscle activation level of the agonist muscle.

In EMG signal recording, the muscle activation level $r_{t}^{\left(l\right)}\left(l=1,2,\cdots ,L\right)$ in each channel was calculated as a percent of MVC (%MVC) simultaneously with the measurement:

$$\begin{array}{c} r_{t}^{\left(l\right)}=\frac{y_{t}^{\left(l\right)}}{y_{\text{max}}^{\left(l\right)}}, \end{array}$$ (17)

where $y_{t}^{\left(l\right)}$ is the rectified and smoothed EMG signal of channel *l* at *t*, and $y_{\text{max}}^{\left(l\right)}$ is the maximum value of pre-measured ${\hat{y}}_{t}^{\left(l\right)}$ during the MVC. The subjects were presented with the muscle activation level of the agonist muscle $r_{t}^{\left(m\right)}$ by using a bar graph in real time (Fig 4). The white vertical line in Fig 4 showed the desired muscle activation level, and the subjects were instructed to perform each motion while maintaining this line. First, the subjects performed and maintained each motion for 10 seconds with the desired muscle activation level $r_{t}^{\left(m\right)}$ at 40%, and recording of the training data was conducted. Next, the task of maintaining each motion for 10 seconds was conducted over 10 trials in two conditions of the desired muscle activation level at 40% and the target muscle activation level at 80%, and recording of the testing data was also conducted. Feature extraction for the training and testing data was then conducted according to the method proposed by Fukuda *et al*. [4], in which the measured EMG signals are rectified and smoothed, and then normalized to make the sum of all the channels equal to 1.0.

In the motion classification task, a neural network called the log-linearized Gaussian mixture network (LLGMN) [4, 25], which can estimate the posterior probability of each class, was used. For LLGMN learning, 100 samples of the artificial EMG signals at 80%MVC, which were generated based on the proposed model, and 100 samples of the measured EMG signals, which were randomly sampled from the training data at 40%MVC, were used for each motion.

To calculate the mean variance ${\bar{\sigma }}_{\left(c,l\right)}^{2}$ of the channel *l* in the motion *c* (*c* = 1, 2, ⋯, *C*) at 80%MVC, Eq (10) was vectorized:

$$\begin{array}{c} {\bar{\sigma }}_{\left(c,l\right)}^{2}={\left(\frac{1-\sum_{i=0}^{N-1}\;a_{i}}{\sum_{i=0}^{N}\;b_{i}}\right)}^{2}\;\frac{\pi }{2}\eta_{\left(c,l\right)}^{2}E{\left[y_{t}^{\left(c,l\right)}\right]}^{2}, \end{array}$$ (18)

where *η*_(*c*,*l*)_ is the proportional gain of the channel *l* in the motion *c*, and is defined as follows:

$$\begin{array}{c} \eta_{\left(c,l\right)}=\frac{0.8}{\lambda_{\left(c,m_{c}\right)}}, \end{array}$$ (19)

where λ_(*c*,*m*_*c*_)_ is the entire time mean value of the muscle activation level of the channel *m*_*c*_ corresponding to the agonist muscle in the motion *c* at 40%MVC. The classification rate was calculated for 1000 samples, sampled from the 5000-th sample to the 6000-th sample of the testing data, and the average classification rate was derived from ten trials.

In the proposed method, 200 samples, including 100 samples of the measured data and 100 samples of the artificially generated data, were used for LLGMN learning, as described above. It is well known that motion classification based on machine learning tends to improve classification accuracy by only increasing the number of training samples in general. For comparison, therefore, the average classification rate was also calculated for two cases: (1) learning conducted with only 100 samples of the measured data at 40%MVC, and (2) learning conducted with 200 samples accumulated by simply increasing the number of samples of the training data at 40%MVC by random sampling.

## Results

### Generation accuracy of the proposed model

**Fig 5:**
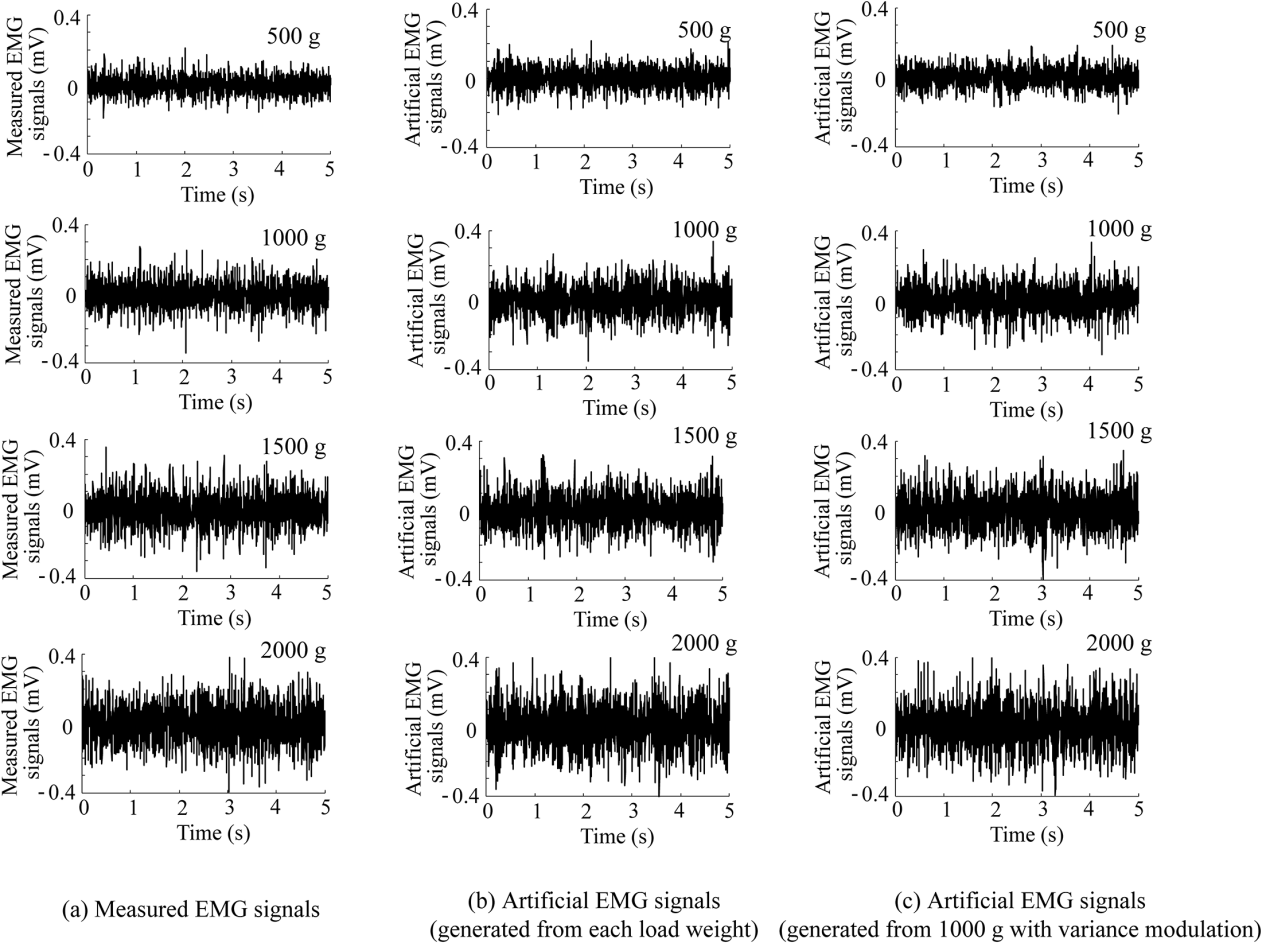
Examples of measured and artificial EMG signals for each load weight. (a) Measured EMG signals. (b) Artificial EMG signals generated from the measured EMG signals under each load weight based on the proposed model. (c) Artificial EMG signals generated from the measured EMG signals under a 1000 g load based on the proposed model with the variance modulation.

Fig 5 shows examples of (a) the measured EMG signals, (b) the artificial EMG signals generated from the measured EMG signals under each load weight based on the proposed model, and (c) the artificial EMG signals generated from the measured EMG signals under a 1000 g load based on the proposed model with the variance modulation, for different load weights: 500, 1000, 1500, and 2000 g.

**Fig 6:**
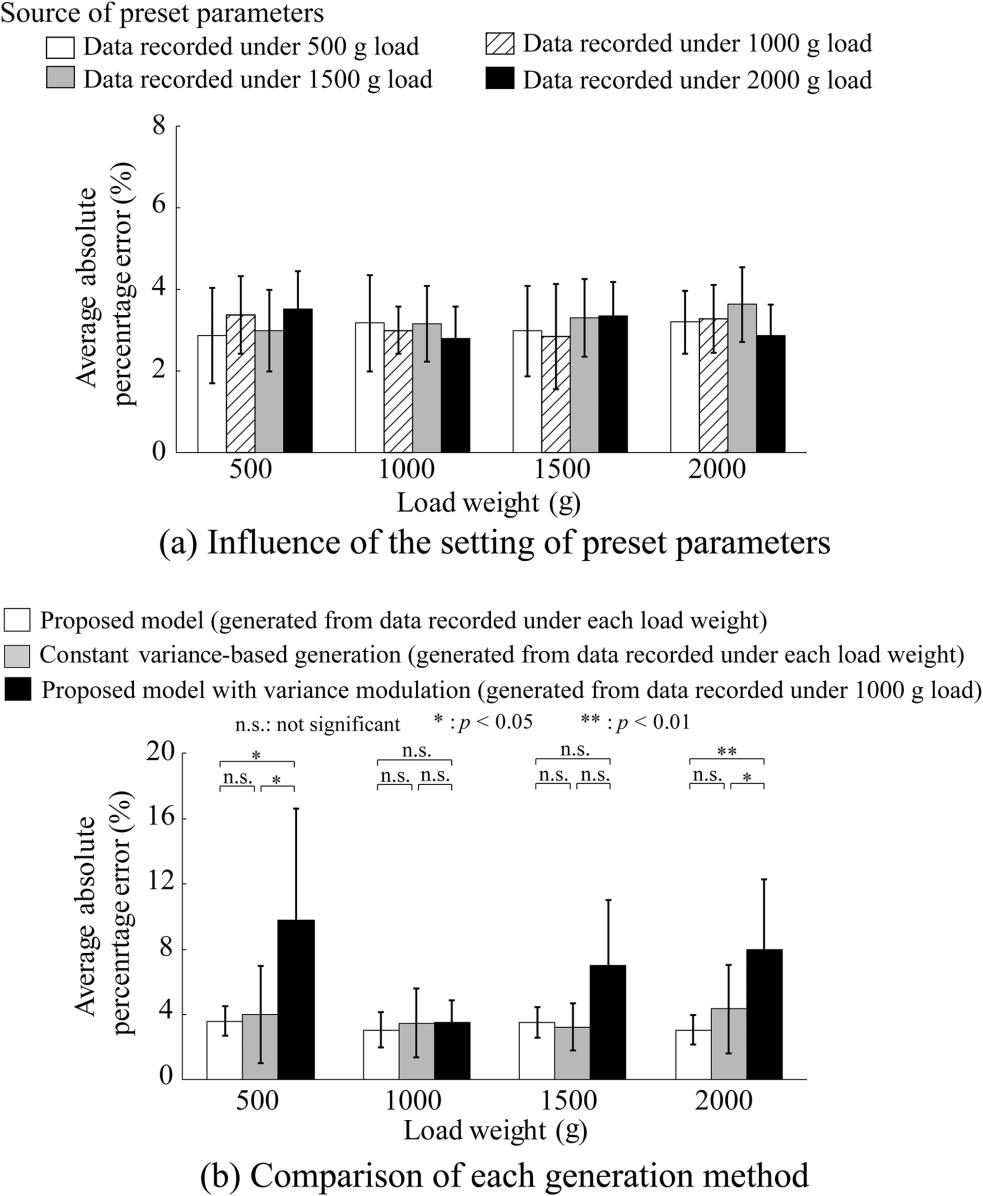
Average absolute percentage error in average amplitude for each load weight. (a) Influence of the recording source of the preset parameters. (b) Comparison of each generation method. Error bars represent the standard deviations for all subjects.

**Fig 7:**
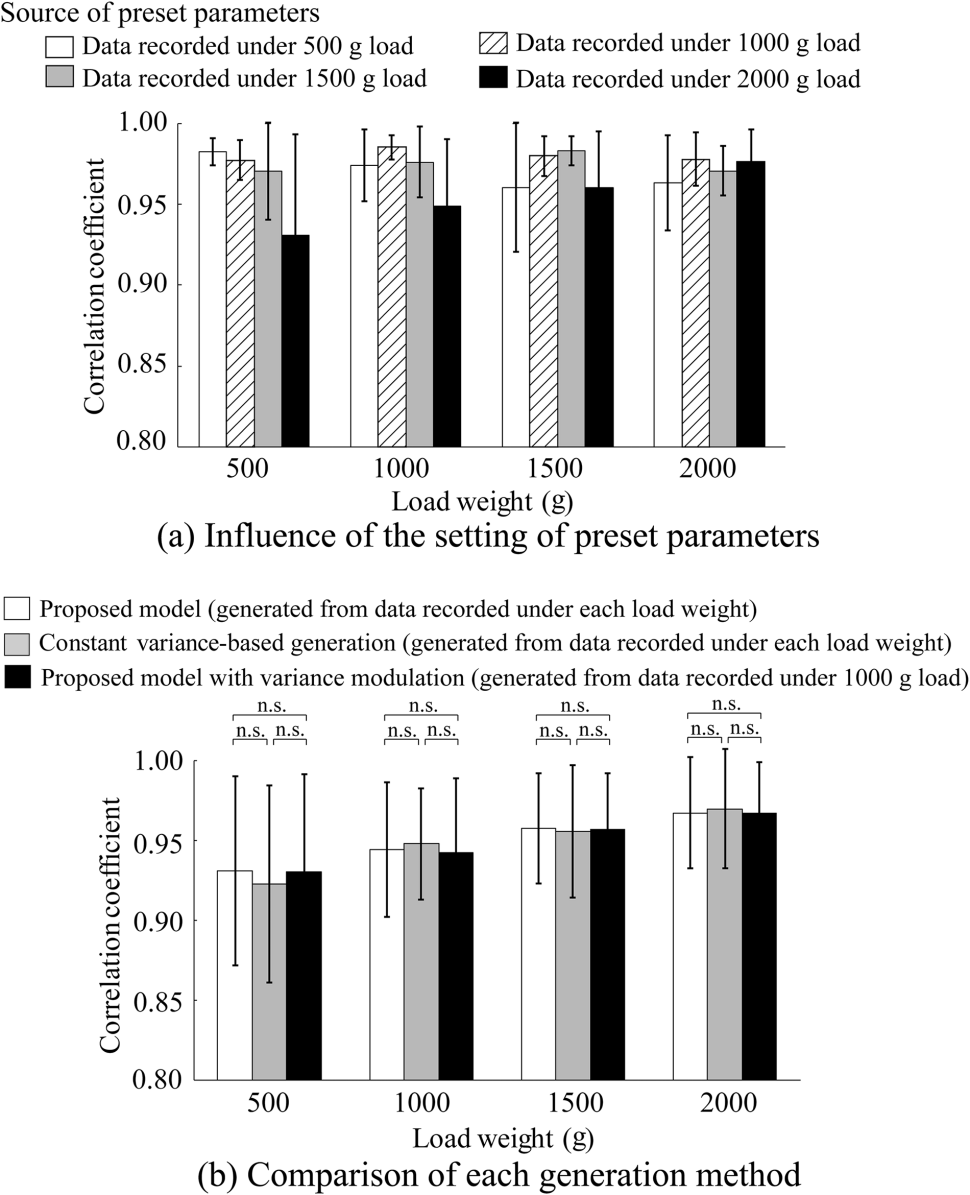
Correlation coefficient in power spectrum for each load weight. (a) Influence of the recording source of the preset parameters. (b) Comparison of each generation method. Error bars represent the standard deviations for all subjects. All correlation coefficients had *p* < 0.001.

**Fig 8:**
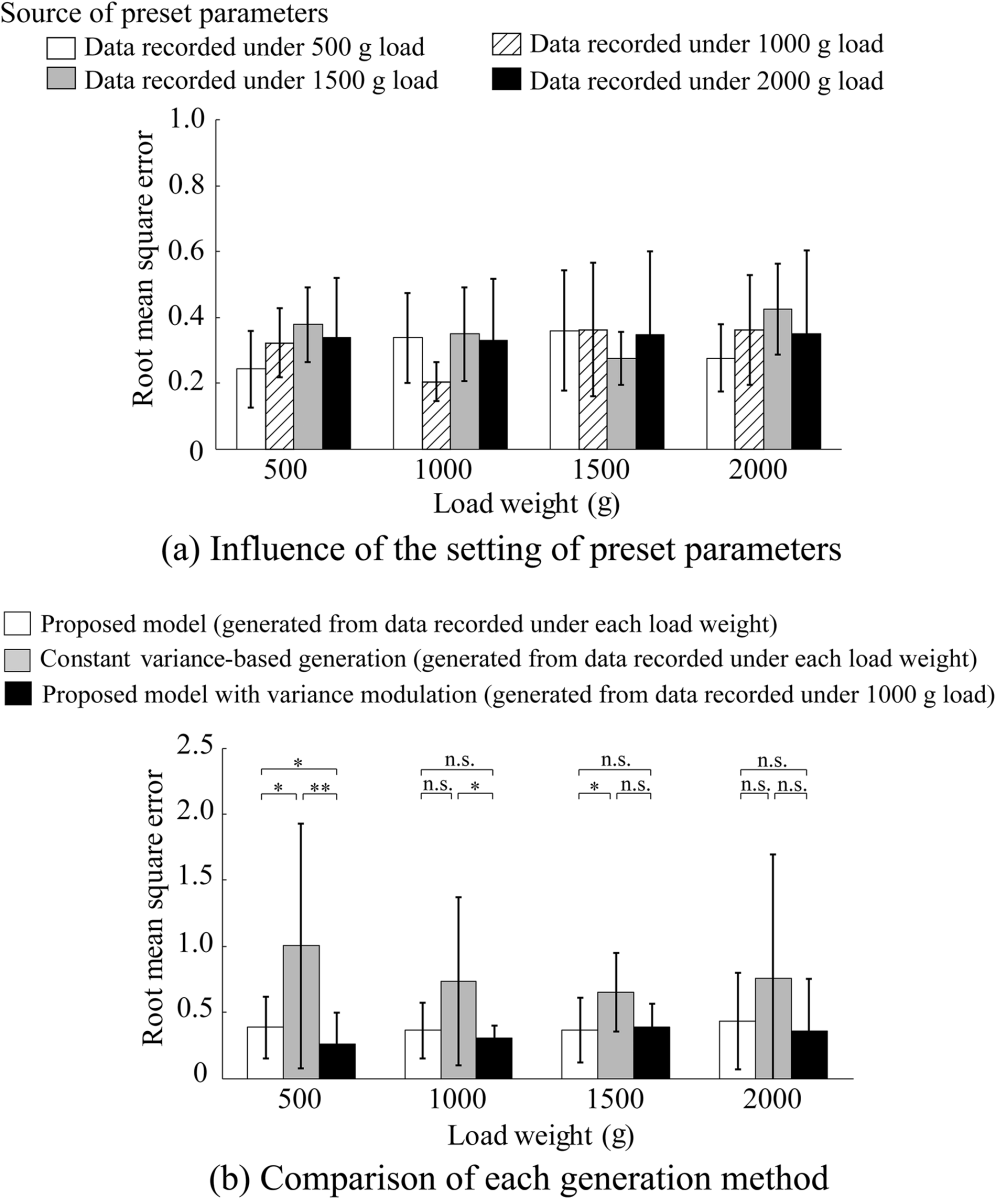
Root mean square error (RMSE) in kurtosis for each load weight. (a) Influence of the recording source of the preset parameters. (b) Comparison of each generation method. Error bars represent the standard deviations for all subjects.

The influence of the recording source of the preset parameters is shown in Figs 6a, 7a and 8a. Fig 6a shows the average absolute percentage error in the average amplitude between the measured and artificial EMG signals based on the proposed model for each load weight. The preset parameters were set using data recorded under 500, 1000, 1500, and 2000 g loads. Fig 7a shows the correlation coefficients in the power spectrum density and Fig 8a shows the RMSE in the kurtosis. The figures show the average values of all the subjects. One-way ANOVA tests (significant level: 0.5%) were performed for each index to detect the influence of the recording source of the preset parameters on the generation accuracy of the proposed model, and there were no significant differences.

The comparison results for the three generation methods are shown in Figs 6b, 7b and 8b. Fig 6b shows the average absolute percentage error in the average amplitude between the measured and artificial EMG signals based on the proposed model for each load weight compared with the constant variance-based method and the proposed model using the variance modulation. Fig 7b shows the correlation coefficients in power spectrum density and Fig 8b shows the RMSE in kurtosis. The figures show the average values of all the subjects. The statistical test results based on the Steel-Dwass method were also given.

### Applicability to motion classification

**Fig 9:**
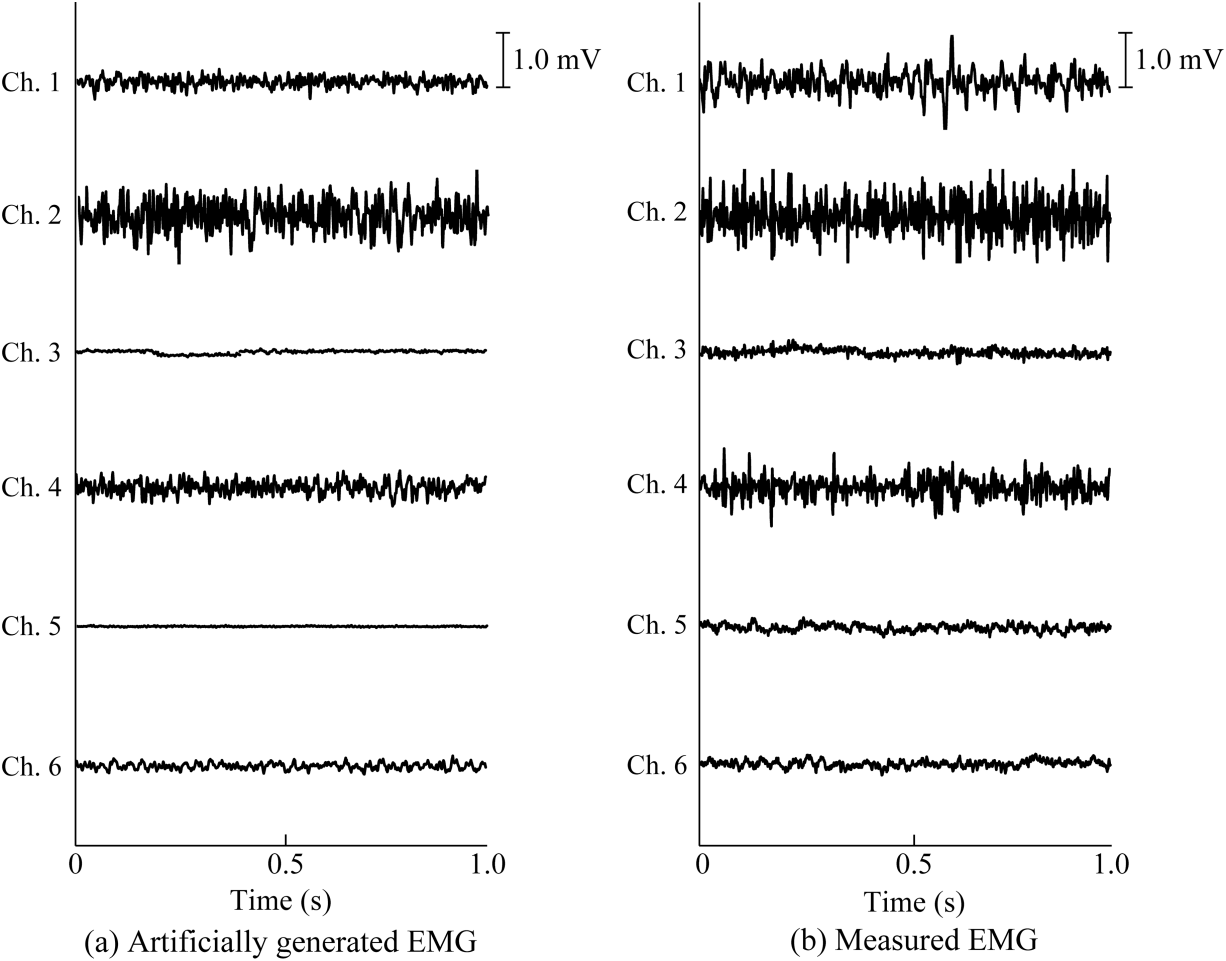
Examples of artificial and measured EMG signals with a muscle activation level of 80%MVC. (a) Artificially generated EMG signals for each channel. (b) Measured EMG signals for each channel. The artificial and measured EMG signals are used for a part of the test and the training data in motion classification, respectively.

Fig 9 shows examples of the measured EMG signals and the artificial EMG signals generated based on the proposed model for a muscle activation level of 80%MVC.

**Fig 10:**
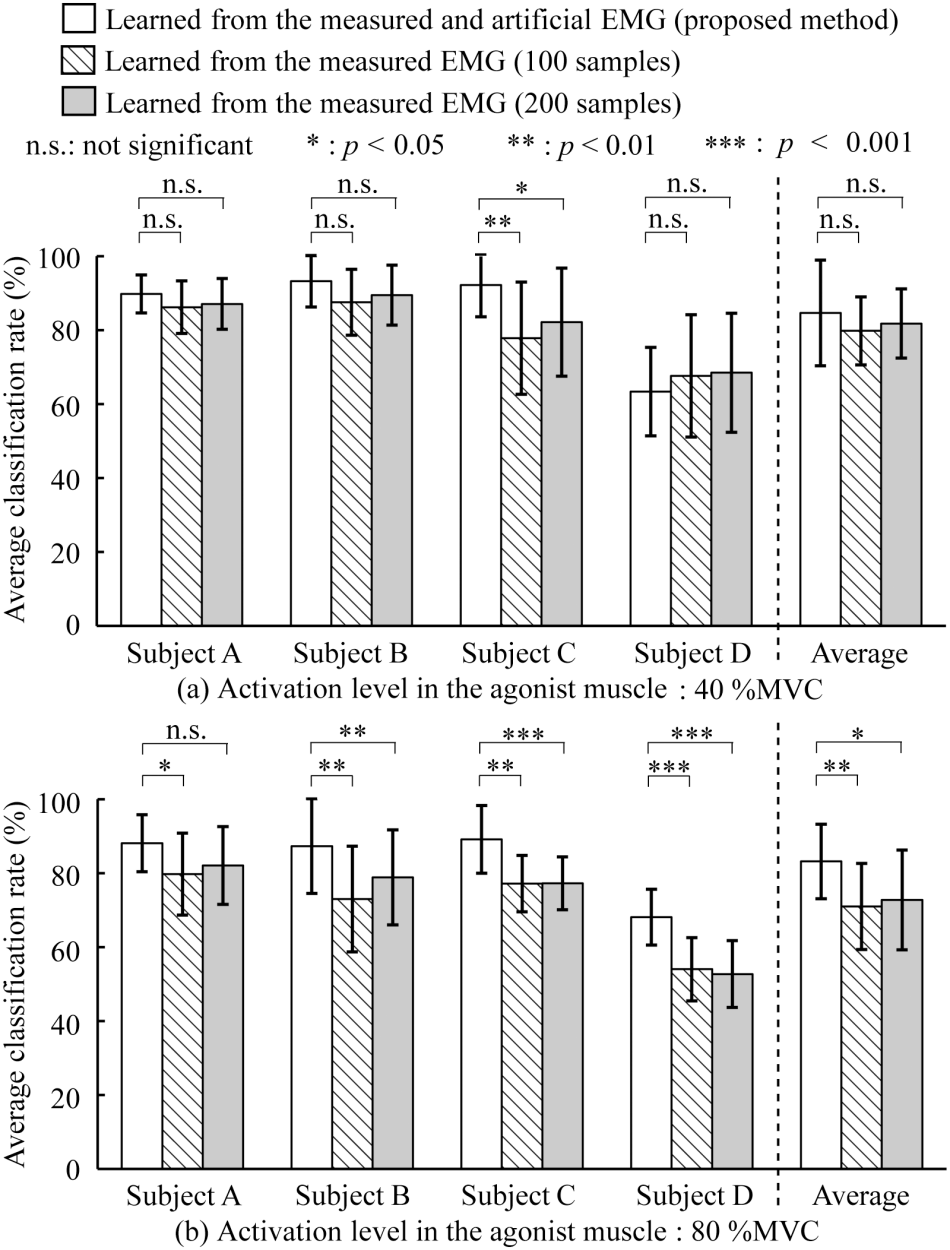
Average classification rates of each method. (a) Muscle activation level of 40%MVC. (b) Muscle activation level of 80%MVC. Error bars in the results of Subjects A–D represent the standard deviations for all trials and those in the average of all subjects represent the standard deviations for all subjects.

Fig 10 shows the average classification rates for each method: (a) muscle activation level of 40%MVC and (b) muscle activation level of 80%MVC. The figure also shows the average classification rates of all the subjects for each muscle activation level. The statistical test results based on the Holm multiple comparison test were also given.

## Discussion

Fig 5 shows that the amplitudes of the measured and artificial EMG signals increased together as the load weights increased. In addition, the amplitudes of the artificial EMG signals are similar to those of the measured EMG signals for each load weight. This result can be confirmed from the average absolute percentage errors in the amplitude shown in Fig 6. In the case where the measured EMG signals were reproduced for each load weight, the error rates in the amplitude of the proposed method are approximately 4% for all sources of the preset parameters and the load weights. No significant differences were found between the results of the proposed method without modulation and those of the constant variance-based method (Fig 6b). However, the standard deviations of the constant variance-based method are larger than those of the proposed method because the accuracy of the variance estimation based on Hayashi *et al*.’s method [13] is better than that of the estimation based on the maximum likelihood method. In the case of using the proportional variance modulation with the gain, the proposed model can predict/generate artificial EMG signals for other load weights with an error rate of 10% or less even if the reproducibility of the EMG amplitude tends to be worse, except for the reference load weight of 1000 g.

Fig 7 shows that the correlation coefficients in the power spectrum densities exhibit strong correlations of over 0.90 regardless of the source of the preset parameters and the load weights for each subject. In addition, no significant differences were confirmed among the three methods because the frequency setting component was the same in each method (Fig 7b). The proposed model therefore can reproduce the frequency component with high accuracy because the frequency components of the proposed model are determined using the AR model given from each subject.

In Fig 8a, no significant differences in RMSE in the kurtosis due to the differences in the recording source of the preset parameters are shown. Fig 8b shows that the RMSE in the kurtosis for the proposed method without/with modulation tends to be lower than that for the constant variance-based method. This result indicates that the artificial EMG signals generated using the proposed model can express the kurtosis of the measured EMG more precisely than those generated by the constant variance-based method. In the case of EMG generation using the constant variance-based method, the artificial EMG follows a Gaussian distribution. However, Hunter *et al*. experimentally showed that the probability density of EMG is more sharply peaked near zero than a Gaussian distribution [26]. Bilodeau *et al*. and Nazarpour *et al*. also reported that the measured EMG density has a larger kurtosis than a Gaussian distribution [27, 28]. In contrast, because the variance is randomly determined from the variance distribution in the proposed model, the artificial EMG signals generated based on the proposed model do not follow a Gaussian distribution. Instead, they follow a distribution with a kurtosis that is more similar to the measured EMG. This indicates that the proposed model based on variance distribution enables artificial EMG signal generation with consideration of the signal-dependent noise affecting fluctuations in the EMG variance value. It should be noted that this representation capability is the most significant point of the proposed model as conventional generation methods cannot generate artificial EMG signals including this noise.

Thus, it is clear that the proposed model can generate artificial EMG signals that reproduce the amplitude, frequency component, and kurtosis of the measured EMG signals. The usability of the proposed artificial EMG generation model is also suggested from the viewpoint of the preset parameter setting because the recording source of the preset parameters does not significantly influence the generation accuracy of the model. Moreover, artificial EMG signals at the arbitrary muscle activation level can be generated from EMG signals recorded under other muscle activation levels with a certain precision if the proportional gain in Eq (10) is appropriately given.

Fig 9 shows that the artificially generated EMG signals based on the proposed model possess features that are close to those of the measured EMG signals at 80%MVC. This result indicates that multi-channel artificial EMG signals with a high muscle contraction level can be generated from pre-measured EMG signals with a low muscle contraction level using the proposed method. In Fig 10a, the testing data for the muscle activation level of 40%MVC show that there are no significant differences between each method in the subjects, except for Subject C, and in the averages of all the subjects. These results suggest that the classification ability of the proposed method is equal to or better than that of every other method when the muscle activation levels of the training and testing data are equal.

By contrast, on the testing data for the muscle activation level of 80%MVC, the proposed method shows significantly higher classification rates than other methods in all the subjects and in their average (Fig 10b). The decreases in the classification rates for the methods, where the measured EMG signals are only learned, can probably be attributed to the increased fluctuation of the EMG patterns of the test data by signal-dependent noise during the strong muscle contraction. Increasing the number of learning samples from the measured signals tends to improve the classification rate. However, the proposed method can be used to generate artificial EMG signals with strong muscle contraction involving noise superimposed onto the EMG depending on the increased muscle force estimated using only the measured EMG signals with low muscle contraction. This facilitates accurate classification without increasing the burden on subjects during the training data collection. These results indicate that the proposed artificial EMG generation model is highly applicable to motion classification via machine learning.

The motion classification experiment conducted in this study was performed only in an offline condition. However, it is known that high classification accuracy in an offline condition yields better classification performance in a real-time context [29]. The classification accuracy in the online condition also tends to be better than that in the offline condition because the subjects can adjust their EMG pattern according to the feedback of the classification results [30]. Therefore, it can be expected that the proposed classification method will also work effectively in an online environment. In addition, the proposed method should have high applicability in a real-time context because it can directly generate artificial EMG signals for training data using measured EMG signals by setting the parameters in advance. On the other hand, because many factors (e.g. sensor movement, sweating, and muscle fatigue) affect classification performance in the online condition, verification of the robustness of the proposed method against such environments will have to be carried out in future studies.

## Conclusion

This paper proposed an artificial EMG generation model based on signal-dependent noise. The proposed model estimates the variance distribution of EMG signals using the inverse gamma distribution, and generates artificial EMG signals with signal-dependent noise superimposed according to muscle activation levels. This is the major distinctive feature of our method compared with existing artificial EMG generation models.

The evaluations conducted on the generated artificial EMG signals and the comparison in terms of amplitude, frequent component, and kurtosis of EMG distribution revealed that the proposed variance distribution-based generation method can reproduce the features of the measured EMG signals during isometric muscle contraction. In the motion classification experiments conducted, the classification rates during strong muscle contraction were improved by using artificial EMG signals for training data. Thus, it is clear that the proposed model can generate artificial EMG signals having similar features to the measured EMG signals by setting suitable variance distribution and frequency characteristics. Moreover, it is possible to effectively apply the proposed model to motion classification.

A limitation of the proposed model and the proposed classification method is an assumption that a linear relationship exists between the muscle activation level and the mean of *σ* in Eqs (10) and (18). Previous studies found that the relationship between the muscle force and the EMG amplitude is sometimes nonlinear [31, 32]. In future research, therefore, it will be necessary to consider this nonlinear relationship for a more accurate estimation and generation of EMG signals during strong muscle activation. Further, we would like to apply the proposed model to control myoelectric hands.
